# What predicts the early sexual debut among unmarried adolescents (10–19 years)? Evidence from UDAYA survey, 2015–16

**DOI:** 10.1371/journal.pone.0252940

**Published:** 2021-06-10

**Authors:** T. Muhammad, Shobhit Srivastava, Pradeep Kumar, Sangram Kishor Patel

**Affiliations:** 1 Department of Population Policies and Programs, International Institute for Population Sciences, Mumbai, Maharashtra, India; 2 Department of Mathematical Demography & Statistics, International Institute for Population Sciences, Mumbai, Maharashtra, India; 3 Population Council, New Delhi, India; University of Southern Queensland, AUSTRALIA

## Abstract

**Introduction:**

The societal norm in India is such that adolescents are expected to respect and follow traditional values and view early sexual debut as undesirable and deviant from the social mores. However, a dramatic shift in attitudes towards sex before marriage has been observed in India. We in this study, aim to study the factors associated with early sexual debut among unmarried adolescents.

**Materials and methods:**

The study used data from the Understanding the lives of adolescents and young adults (UDAYA) survey conducted in 2016 with 15,388 adolescents aged 10–19 years from two Indian states. Bivariate and logistic regression analyses were performed to determine the associated factors.

**Results:**

Adolescent boys (9%) were more prone to early sexual debut compared to girls (4%). Both boys (17.2%) and girls (6%) who were school dropouts had significantly higher chances of early sexual debut. Boys who had rare [OR: 2.28; CI: 1.12–4.64] or frequent media exposure [OR: 2.70; CI: 1.36–5.32] were significantly more likely to report early sexual debut than those who had no media exposure. Further, the likelihood of early sexual debut was significantly higher among boys [OR: 3.01; CI: 2.34–3.87] and girls [OR: 1.87; CI: 1.12–3.12] who had exposure to pornography compared to their counterparts. The odds of early sexual debut were higher among boys [OR: 1.89; CI: 1.19–3.01] and girls [OR: 1.77; CI: 1.30–2.41] who had moderately-severe/severe depressive symptoms compared to their counterparts.

**Conclusions:**

The results highlight that Indian unmarried adolescents demand the appropriate knowledge to promote safer sexual behavior and lead a responsible and healthy lifestyle. The preventive efforts must be multifaceted with involvement at the individual and parental levels. Especially, interventions appear advantageous to be parents-focused emphasizing family life education that can prevent risky sexual behaviors among adolescent boys and girls. And the public programs should focus on sexual health promotion considering the physical and psychosocial changes during early ages of sex life.

## Introduction

Due to the recent changes in lifestyle as a result of civilization and urbanization, the sexual and reproductive health of adolescents has become increasingly at stake [1]. These individuals in their second decade of life in terms of their health risks are considered as a unique group [2]. In India, where the adolescent population between age 10 and 19, constitutes 20 percent of the population [3], premarital sexuality during this period has become more or less accepted breaking the long-prevailing traditional norms and customs against it [4].

In the context of technological advancements, media exposure such as television, mobile telephones, and magazines, was found as a significant factor associated with premarital sex among adolescents [5]. The adolescents have an increased opportunity to attend the sex-related mainstream media and abundant sexual materials that are proven to be influential on their sexual attitudes and behaviors [6]. A study in India found that the adolescents who are studying in vernacular schools, accessing pornography, and having an unfriendly relationship with parents had a higher likelihood of sex initiation [7]. Also, the mainstreaming of pornography over the last decades created a favorable environment for early sexual experiments among adolescents. A plethora of studies have found an association between pornography viewing and premarital sex [8, 9].

Furthermore, a review of 35 recent longitudinal studies found that the onset of intercourse was more strongly associated with alcohol use, misconduct, school problems for boys, and depressive symptoms for girls [10]. A study that specifically examined the predictors of sexual debut at age 16 or younger found that variables including alcohol dependence, conduct disorders, and other familial background were significantly associated with increased risk [11]. It may reason out as alcohol drinking decreases the self-control and leads to risky behaviors such as early sexual intercourse [12]. Adolescents who reported smoking habits were also more likely to have initiated sex early [7]. Studies in low and middle-income countries also revealed that adolescents who reported, substance use and living out of parental supervision were associated with early sexual debut [13–15].

Similarly, children who experienced sexual violence were found to be at increased risk of perpetrating sexual coercion and reporting early sexual debut [16]. Sahay et al. found that the adolescents who reported sexual abuse were at increased risk for early sex initiation [7]. Moreover, the family background was found, contributing most to premarital sexual activities [17]. The study showed that changes in maternal and paternal co-residence have implications for the timing of sexual initiation for adolescents [18]. While parents were viewed as important sources of information for sexual and reproductive health, the study found that the female adolescents who were raised by relatives were less likely to report sex debut compared to those raised by their parents [19]. Further, living with friends or relatives living alone without parental control was found to be significantly associated with premarital sexual practices [20].

The societal norm in India is such that adolescents are expected to respect and follow traditional values and view premarital sex as undesirable and deviant from the social mores [4]. Previous studies in low-income countries found that the students who had reported early sexual debut are exposed to risky sexual behaviors such as unprotected sexual intercourse, unwanted pregnancies, unsafe abortion, and several psychosocial problems [21, 22]. Literature also suggests that the attitudes and sexual behavior of adolescents are usually influenced by demographic and socioeconomic characteristics, especially in a country where expressed social norms often condemn premarital sex [23–25]. Moreover, there are large gender-based differences in sexual conduct among unmarried adolescents [26].

Though a dramatic shift in attitudes towards sex before marriage has been observed in India, the factors associated with early sexual debut are poorly explored. Given the paucity of such research, we aim to study the determinants and risk factors of early sexual debut among unmarried adolescents in two large states (Uttar Pradesh and Bihar) of India. In the present study, we hypothesize that:

H1: Exposure to media and pornography are related to the early sex debut among unmarried adolescents.
H2: Mental distress and substance use are positively associated with the initiation of sexual intercourse at early ages.
H3: Sexual and parental abuse is a risk factor for early sexual debut among unmarried adolescents.
H4: The presence of parents at home is a protective factor against early sexual debut among adolescents.
H5: Current schooling status is a predictor of early sexual debut among unmarried adolescents.

## Materials and methods

### Data

This study archived data from the Understanding the lives of adolescents and young adults (UDAYA) project survey, which was conducted in two Indian states Uttar Pradesh and Bihar, in 2016 by Population Council under the guidance of the Ministry of Health and Family Welfare, Government of India. The survey collected detailed information on family, media, community environment, assets acquired in adolescence, and quality of transitions to young adulthood indicators. The UDAYA adopted a multi-stage systematic sampling design to provide the estimates for states well as urban and rural areas of the states. The sample size for Uttar Pradesh and Bihar was 10,350 and 10,350 adolescents aged 10–19 years, respectively. The effective sample size for this study was 15,388 adolescents (boys-5,969 and girls-9,419) who were unmarried at the time of the survey [27].

#### Ethics considerations

The Population Council Institutional Review Board provided ethical approval for the survey. Adolescents provided individual written consent to participate in the study, along with a parent/guardian for unmarried adolescents younger than 18 years. The Population Council identified referral services for counseling and health services to offer respondents if necessary, and fieldworkers were trained on ethical issues and sensitivity. In addition, interviewing boys and girls in separate segments helped minimize issues related to confidentiality and response bias.

### Variable description

#### Outcome variable

The early sexual debut was coded using the variable age at first sex among unmarried adolescents. Adolescents who experienced their first intercourse before 18 years of age were coded as 1 “early sexual debut” and 0 “otherwise” [22].

#### Explanatory variable

*Individual factors*. Media exposure was coded as “no exposure”, “rare exposure” and frequent exposure”. Media exposure contains exposure to television, radio, and newspaper. Exposure to pornographic material or blue films was coded as “no” and “yes”. Depressive symptoms were assessed by asking nine questions from the respondents; the respondent was asked about the symptoms for the past two weeks only. The questions included, a. had trouble falling asleep or sleeping too much, b. feeling tired or having little energy, c. poor appetite or eating too much, d. trouble concentrating on things, e. had little interest or pleasure in doing things f. feeling down, depressed or hopeless, g. feeling bad about yourself, h. been moving or speaking slowly, i. had thoughts that respondent would be better off dead. All the above questions were asked on a scale of four, i.e., 0 “not at all”, 1 “less than one week”, 2 “one week or more”, and 3 “nearly every day”. The scale of 27 points was then generated using egen command in STATA 14. (Cronbach alpha: 0.86) [28]. The variable was then coded into three categories, i.e., a. Mild/minimal (0–9), b. Moderate (10–14) and c. moderately severe/severe (15–27). Substance use included consumption of tobacco products and alcohol, which was coded as “no” and “yes”. Sexual violence and parental violence among adolescents was coded as “no” and “yes”. Parent’s co-residence with the adolescent was coded as “both parents co-reside”, “anyone parent co-reside” and “no one co-reside”. Current schooling status was coded as “never attended”, “Dropout” and “currently attending”. Working status was recoded as “no” and “yes”.

*Household factors*. Caste was coded as “Scheduled Caste/Scheduled Tribe (SC/ST)” and “non-SC/ST” [29]. The “non-SC/ST” caste includes Other Backward Class and Others. The Scheduled Caste includes “untouchables”, a group of the population that is financially/economically and socially segregated by their low status as per Hindu caste hierarchy. The Scheduled Castes (SCs) and Scheduled Tribes (STs) are among the most disadvantaged socio-economic groups in India. The OBC is the group of people who were identified as “educationally, economically and socially backwards”. The OBCs are considered low in the traditional caste hierarchy but are not considered untouchables. The “other” caste category is identified as having higher social status [30]. Religion was coded as “Hindu” and “non-Hindu”. Wealth index was coded as “poorest” “poorer” middle” richer” and richest”. Residence was available in the data as “urban” and “rural”. Survey was conducted in two states “Uttar Pradesh” and “Bihar”.

### Statistical analysis

Descriptive statistics, along with bivariate analysis, was done to examine the preliminary results. For analyzing the association between the binary outcome variable and other explanatory variables, the binary logistic regression method [31] was used. The outcome variable was early sexual debut among unmarried adolescents aged 10–19 years.

The equation for logistic distribution is

$$ln\left(\frac{\pi }{1-\pi }\right)=\alpha +\beta_{1}X_{1}+\beta_{2}X_{2}+\beta_{3}X_{3}\ldots \ldots .\beta_{n}X_{n}$$

Where, *β*_0_,…..,*β_M_*, are regression coefficients indicating the relative effect of a particular explanatory variable on the outcome variable. These coefficients change as per the context in the analysis in the study. The Variance inflation factor (VIF) [32, 33] was estimated to check multicollinearity in the variables used and it was found that there was no evidence of multicollinearity.

## Results

**Fig 1:**
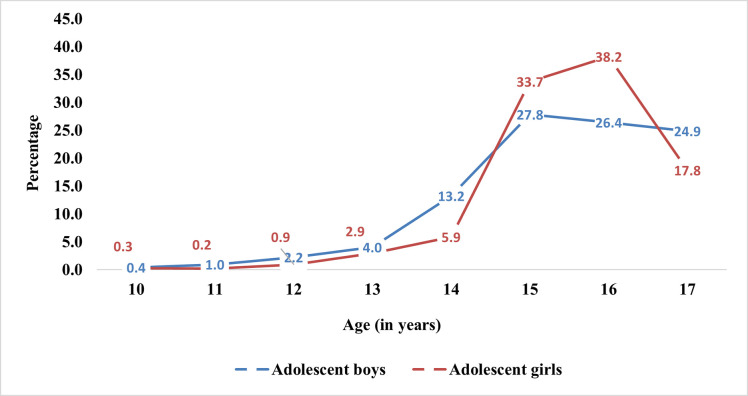
Early premarital sexual debut among adolescent boys and girls according to their age.

Fig 1 depicts that the percentage of adolescents who had early premarital sexual debut increases with an increase the age irrespective of gender. Adolescent boys had a higher early sexual debut than girls till age 14 years, and after that, it was decreasing. Moreover, it was highest among adolescent girls who were 15 (33.7 percent) and 16 years (38.2 percent) old compared to boys of same age (27.8 percent and 26.4 percent).

Socio-demographic profile of adolescent boys and girls are presented in Table 1. Nearly three-fourth of adolescent boys and six in every ten girls had frequent media exposure. About 12 percent of adolescent boys and only two percent of girls had exposure to pornography, three percent of boys and seven percent of girls had moderately severe/severe depressive symptoms, and 16 percent of boys and two percent of girls used any substances. About 58 percent of adolescent boys and 35 percent of adolescent girls experienced parental violence. A higher proportion of adolescents lived with their parents (boys-82.8% and girls-81.5%), and the majority of adolescent boys (78.5%) and girls (66.1%) were currently attending school. Nearly 27 percent of boys and 20 percent of girls were working.

**Table 1:** Socio-demographic profile of adolescents boys and girls aged 10–19 years.

Background characteristics	Adolescent boys		Adolescent girls	
	Sample	Percentage	Sample	Percentage
**Individual factors**				
**Media exposure**				
No exposure	335	5.6	1,345	14.3
Rare	1,078	18.1	2,539	27.0
Frequent	4,555	76.3	5,535	58.8
**Exposure to pornography**				
No	5,273	88.4	9,253	98.2
Yes	696	11.7	166	1.8
**Depressive symptoms**				
Mild/minimal	5,541	92.8	8,105	86.1
Moderate	255	4.3	621	6.6
Moderately severe/severe	173	2.9	692	7.4
**Substance use**				
No	4,990	83.6	9,271	98.4
Yes	979	16.4	148	1.6
**Sexual violence**				
No	5,862	98.2	8,753	92.9
Yes	107	1.8	666	7.1
**Parental violence**				
No	2,489	41.7	6,143	65.2
Yes	3,480	58.3	3,276	34.8
**Parents co-residence**				
Both co-reside	4,940	82.8	7,672	81.5
Any one co-reside	870	14.6	1,489	15.8
No one co-reside	160	2.7	258	2.7
**Current schooling status**				
Never attended	190	3.2	639	6.8
Dropout	1,092	18.3	2,559	27.2
Currently attending	4,687	78.5	6,221	66.1
**Working status**				
No	4,377	73.3	7,582	80.5
Yes	1,592	26.7	1,837	19.5
**Household factors**				
**Caste**				
SC/ST	1,605	26.9	2,241	23.8
Non-SC/ST	4,364	73.1	7,178	76.2
**Religion**				
Hindu	5,024	84.2	7,234	76.8
Non-Hindu	945	15.8	2,185	23.2
**Wealth index**				
Poorest	704	11.8	1,213	12.9
Poorer	1,193	20.0	1,666	17.7
Middle	1,374	23.0	1,966	20.9
Richer	1,391	23.3	2,315	24.6
Richest	1,308	21.9	2,259	24.0
**Residence**				
Urban	1,030	17.3	1,625	17.3
Rural	4,939	82.7	7,794	82.7
**State**				
Uttar Pradesh	4,069	68.2	6,637	70.5
Bihar	1,900	31.8	2,782	29.5
Total	5,969	100.0	9,419	100.0

SC/ST: Scheduled Caste/Scheduled Tribe.

Percentage of adolescent boys and girls who had early premarital sexual debut by background characteristics are shown in Table 2. Overall, adolescent boys (9%) had higher early premarital sexual debut compared to adolescent girls (4%). It was significantly higher among both boys (22.3%) and girls (6%) who had pornography exposure than those who did not have. Moreover, adolescent boys (22.4%) and girls (8.4%) who had moderately severe/severe depressive symptoms reported higher prevalence of early sexual debut. Similarly, both adolescent boys (23.8%) and girls (10.4%) who used any substances had significantly higher prevalence of early sexual debut than those who did not use. Interestingly, both boys (17.2%) and girls (6%) who were school dropout had significantly higher early premarital sexual debut than those who were currently attending school (6.9% and 3.5%). The early premarital sexual debut was significantly higher among working boys (16.6%) and girls (7.8%) compared to those who were not working. Adolescent boys (11.3%) and girls (7.7%) who belonged to SC/ST categories reported a higher percentage of early sexual debut compared to non-SC/ST adolescents. Moreover, both boys (9.8%) and girls (4.4%) who lived in rural areas had a significantly higher prevalence of early premarital sexual debut than those who lived in urban areas.

**Table 2:** Percentage distribution of unmarried adolescents who had early sexual debut by background characteristics.

Background characteristics	Adolescent boys		Adolescent girls	
	%	p-value	%	p-value
**Individual factors**				
**Media exposure**		0.124		0.001
No exposure	4.8		4.8	
Rare	8.3		5.2	
Frequent	9.5		3.5	
**Exposure to pornography**		0.001		0.001
No	7.3		4.1	
Yes	22.3		6.0	
**Depressive symptoms**		0.001		0.001
Mild/minimal	8.3		3.5	
Moderate	15.0		7.3	
Moderately severe/severe	22.4		8.4	
**Substance use**		0.001		0.001
No	6.1		4.1	
Yes	23.8		10.4	
**Sexual violence**		0.001		0.001
No	8.7		2.9	
Yes	24.9		20.7	
**Parental violence**		0.041		0.001
No	9.3		3.1	
Yes	8.8		6.1	
**Parents co-residence**		0.052		0.046
Both parents co-reside	9.1		4.0	
Any one parent co-reside	8.5		5.0	
No one co-reside	8.6		3.8	
**Current schooling status**		0.001		0.001
Never attended	13.7		3.4	
Dropout	17.2		6.0	
Currently attending	6.9		3.5	
**Working status**		0.001		0.001
No	6.3		3.3	
Yes	16.6		7.8	
**Household factors**				
**Caste**		0.001		0.001
SC/ST	11.3		7.7	
Non-SC/ST	8.2		3.1	
**Religion**		0.454		0.020
Hindu	9.1		4.5	
Non-Hindu	8.8		2.9	
**Wealth index**		0.001		0.001
Poorest	8.3		5.1	
Poorer	9.8		7.1	
Middle	10.5		4.5	
Richer	7.8		2.9	
Richest	8.5		2.5	
**Residence**		0.001		0.001
Urban	5.1		2.8	
Rural	9.8		4.4	
**State**		0.001		0.188
Uttar Pradesh	9.8		4.1	
Bihar	7.4		4.3	
**Total**	9.0		4.2	

SC/ST: Scheduled Caste/Scheduled Tribe; p-value based on chi-square test; %: percentage.

Estimates from logistic regression analysis for early sexual debut among unmarried adolescent boys and girls aged 10–19 years are presented in Table 3. Adolescent boys who had rare [OR: 2.28; CI: 1.12–4.64] or frequent media [OR: 2.70; CI: 1.36–5.32] exposure were 2.28 and 2.70 times significantly more likely to have early sexual debut respectively than those who had no media exposure. The likelihood of early premarital sexual debut was 3.01 and 1.87 times significantly higher among adolescent boys [OR: 3.01; CI: 2.34–3.87] and girls [OR: 1.87; CI: 1.12–3.12] who had exposure to pornography respectively compared to those who did not have exposure. The odds of early sexual debut was 89 percent and 77 percent higher among boys [OR: 1.89; CI: 1.19–3.01] and girls [OR: 1.77; CI: 1.30–2.41] who had moderately severe/severe depressive symptoms compared to those who had mild/minimal symptoms. Similarly, adolescent boys [OR: 2.85; CI: 2.24–3.63] who used any substances were 2.85 times more likely to have early sexual debut than those who did not use any substances.

**Table 3:** Logistic regression estimates for early sexual debut among unmarried adolescents by background characteristics.

Background characteristics	Adolescent boys	Adolescent girls
	OR (95% CI)	OR (95% CI)
**Individual factors**		
**Media exposure**		
No exposure	Ref.	Ref.
Rare	2.28*(1.12,4.64)	1.28(0.88,1.88)
Frequent	2.70*(1.36,5.32)	1.01(0.68,1.48)
**Exposure to pornography**		
No	Ref.	Ref.
Yes	3.01*(2.34,3.87)	1.87*(1.12,3.12)
**Depressive symptoms**		
Mild/minimal	Ref.	Ref.
Moderate	0.96(0.59,1.55)	1.44(1.00,2.07)
Moderately severe/severe	1.89*(1.19,3.01)	1.77*(1.30,2.41)
**Substance use**		
No	Ref.	Ref.
Yes	2.85*(2.24,3.63)	1.47(0.81,2.67)
**Sexual violence**		
No	Ref.	Ref.
Yes	3.08*(1.78,5.31)	6.35*(4.98,8.09)
**Parental violence**		
No	Ref.	Ref.
Yes	1.03(0.83,1.28)	1.59*(1.27,2)
**Parents co-residence**		
Both parents co-reside	Ref.	Ref.
Any one parent co-reside	1.00(0.75,1.34)	1.24(0.94,1.63)
No one co-reside	1.24(0.69,2.23)	0.83(0.42,1.67)
**Current schooling status**		
Never attended	Ref.	Ref.
Dropout	0.99(0.6,1.65)	1.53(0.95,2.47)
Currently attending	0.56*(0.33,0.95)	1.06(0.65,1.72)
**Working status**		
No	Ref.	Ref.
Yes	1.69*(1.30,2.18)	1.43*(1.10,1.86)
**Household factors**		
**Caste**		
SC/ST	Ref.	Ref.
Non-SC/ST	0.77*(0.60,0.99)	0.65*(0.50,0.84)
**Religion**		
Hindu	Ref.	Ref.
Non-Hindu	0.96(0.71,1.3)	1.01(0.75,1.36)
**Wealth index**		
Poorest	Ref.	Ref.
Poorer	1.15(0.76,1.75)	1.7*(1.15,2.49)
Middle	1.31(0.88,1.96)	1.07(0.71,1.61)
Richer	1.04(0.68,1.58)	0.97(0.64,1.47)
Richest	1.12(0.71,1.76)	0.82(0.51,1.31)
**Residence**		
Urban	Ref.	Ref.
Rural	2.39*(1.86,3.07)	1.24(0.95,1.6)
**State**		
Uttar Pradesh	Ref.	Ref.
Bihar	0.83(0.66,1.04)	1.13(0.9,1.42)

OR: Odds ratio; CI: Confidence Interval; Ref: Reference category; SC/ST: Scheduled Caste/Scheduled Tribe

*if p<0.05.

Interestingly, both boys and girls who experienced sexual violence were 3.08 and 6.35 times respectively more likely to have early premarital sexual debut than those who did not experience it. With reference to adolescent boys who never attended school, the early sexual debut was 44 percent less likely among adolescent boys who were currently attending school [OR: 0.56; CI: 0.33–0.95]. Moreover, the odds of the early sexual debut were 69 percent and 43 percent more likely among working boys [OR: 1.69; CI: 1.30–2.18] and girls [OR: 1.43; CI: 1.10–1.86] respectively compared to their counterparts. With reference to SC/ST adolescent boys and girls, the likelihood of early sexual debut was 33 percent and 35 percent less likely among adolescent boys [OR: 0.77; CI: 0.60–0.99] and girls [OR: 0.65; CI: 0.50–0.84] who belonged to non-SC/ST category respectively. Adolescent boys who lived in rural areas were 2.39 times more likely to have early sexual debut [OR: 2.39; CI: 1.86–3.07] than those who lived in urban areas. A similar result was found for adolescent girls, but it was not significant.

## Discussion

Although the sex before marriage has not become socially acceptable in India, the present study shows that the proportion of unmarried adolescents initiating sexual intercourse before age 18 is considerably high. The bivariate analysis showed that some of the individual, family and background characteristics are significantly associated with early initiation of sex among adolescents. The risk factors varied by gender and with different socioeconomic settings. The findings from an Indian study also suggested that social norms are stricter for females against premarital sexual relationships and gender differences in attitudes towards sexuality are still prevailing in the country [26].

Empirical research, especially from developed countries showed that a wide range of factors influences the sexual debut among adolescent boys and girls. Many researchers are of the view that adolescents are naturally prone to increased urge for sexual activities. Further, the increased gap between puberty and marriage has created a longer duration in which young people engage in experimentation with sex and premarital sexual intercourse [34]. In a study, it was revealed that watching sexual depictions or exposure to television that talk about sex predicts and may hasten adolescent sexual initiation [35]. In concordance with this, exposure to media has been found in the present study as a predictor of the onset of early sexual intercourse among adolescent boys.

The finding that shows that watching pornographic video is a risk factor for an early sexual debut is also consistent with previous studies [36, 37]. Further, the findings have also found a linkage of adolescent use of pornography that depicts violence with sexually aggressive behaviors [8]. Understanding the effects of watching pornography on adolescent sexual behavior requires further investigation. Since adolescents who watch pornography could be vulnerable to problematic behavior, there is an urgent need for educating them on the potential negative consequences. However, despite the positive influence of sex education towards promoting overall health and well-being of adolescents, study found greater resistance from various Indian states in incorporating it in the curriculum [38].

Again, the studies of adolescent sexual behavior that examined the influence of mental distress found that the chances of early sexual debut among adolescents increased when there was an increase in the number of psychosocial indicators such as depression [39]. The present analysis also found that both boys and girls who reported depressive symptoms or substance use were at higher risk for having sex. Results from other studies also indicated that adolescents who smoked or used alcohol were more likely than those who had not engaged in such behaviors to have early sex debut [40]. Consistently, adolescent boys and girls in our study who reported substance use were more likely to debut sexual intercourse at less than age 18. The connection between early sexual activities, psychosocial distress, and substance use indicates that prevention programs should broaden sexual health promotion that includes awareness on healthy sexual decision-making among adolescents.

Evidence suggests that adolescents initiating sex early might have experienced any kind of sexual violence during early ages and they may have future repercussions. For instance, a study in India indicated that adolescent participants who reported a history of physical or sexual violence were practicing higher sexual risk behaviors than their counterparts [41]. The results of the present study also have shown that the adolescent boys and girls who experienced sexual violence or abuse from their parents had significantly higher chances of an early sexual debut than their counterparts. Consistently, a study in South Africa found that the female adolescents who were exposed to any violence were more likely to have early sexual debut compared to those who were not abused [19]. Furthermore, family structure and parental attitudes toward sexuality play an important role in sexual behaviors among adolescents [42]. It is found that the absence of both parents from the household was associated with an earlier sexual debut. Results show that the adolescent boys were more likely to report early sex debut if they co-resided with neither of their parents. This is consistent with previous studies that have underscored the importance of the physical presence of parents on adolescent sexual behavior, reproductive health, and well-being [43–45].

Previous studies conducted in several Sub-Saharan African countries have shown that young people enrolled in school are less likely than those not in school to report sexual activity [46]. The analysis of current schooling status found that the odds of early sexual debut among adolescent boys who are currently attending school were significantly lower. Similar evidence is found that showed adolescent boys who are enrolled in school are less likely than those of the same age who are not enrolled to engage in premarital sex, regardless of the level of enrolment [47]. Additionally, given the risk of pregnancy among adolescent girls, early sexual initiators are more likely to drop out of school thereby limiting their educational and vocational futures as the evidence indicates from low and middle-income countries [48]. Hence, causality in this regard cannot be drawn from our study. Nonetheless, in consistence with other studies, for adolescents in our study, currently attending school (for boys), living with both parents and higher socioeconomic status were protective against early sexual relations [49, 50].

The limitation of the present study is that many related factors associated with early initiation of sexual intercourse among adolescents such as physical maturity, attitudes towards sex, misconduct, school problems, dating behavior, parental communication and characteristics of the partner were not examined. Furthermore, this study was based on a data collected in the cross-sectional survey, and therefore, the causality between the associated factors in the study cannot be drawn. However, apart from that, the study has its strength, i.e., the depth of questions asked are relatable to the available research work. Moreover, Uttar Pradesh and Bihar are the two states with the highest proportion of adolescents in India [51], therefore representing the reliable and valid situation of Indian adolescents in less developed states. Further, Bihar is the second state that contributes the highest adolescents (9.2%) and youth population (7.6%) in the total country’s population [51].

## Conclusion

Given the recent changes in attitudes towards early sex debut among adolescents in India, the present study found that the correlates of such sexual behaviors included in consistence with other studies, the exposure to media and watching pornography, depressive symptoms and substance use, sexual and parental abuse, parental absence at home (lack of connectedness and supervision), never attended schooling status and lower socioeconomic status.

The results highlight that Indian unmarried adolescents demand the appropriate knowledge to promote safer sexual behavior and lead a responsible and healthy lifestyle. Hence, the preventive efforts must be multifaceted with involvement at the individual as well as parental levels. Especially, interventions appear advantageous to be parents-focused emphasizing family life education that can prevent risky sexual behaviors among adolescent boys and girls. And the public programs should focus on sexual health promotion considering the physical and psychosocial changes during early ages of sex life. Further, future research attention is warranted on this potentially vulnerable subpopulation regarding the determinants and consequences of their early premarital transition to the sexual experience.
